# Supplementary material for: Regulation and Therapeutic Targeting of MTHFD2 and EZH2 in KRAS-Mutated Human Pulmonary Adenocarcinoma
Source: Metabolites. 2022 Jul 15;12(7):652. doi: 10.3390/metabo12070652 (PMC9324032; doi:10.3390/metabo12070652)
Supplement: Supplementary file 1 [file metabolites-12-00652-s001.zip › Li_et_al_Supplementary_Figure S1.pdf]

Figure S1

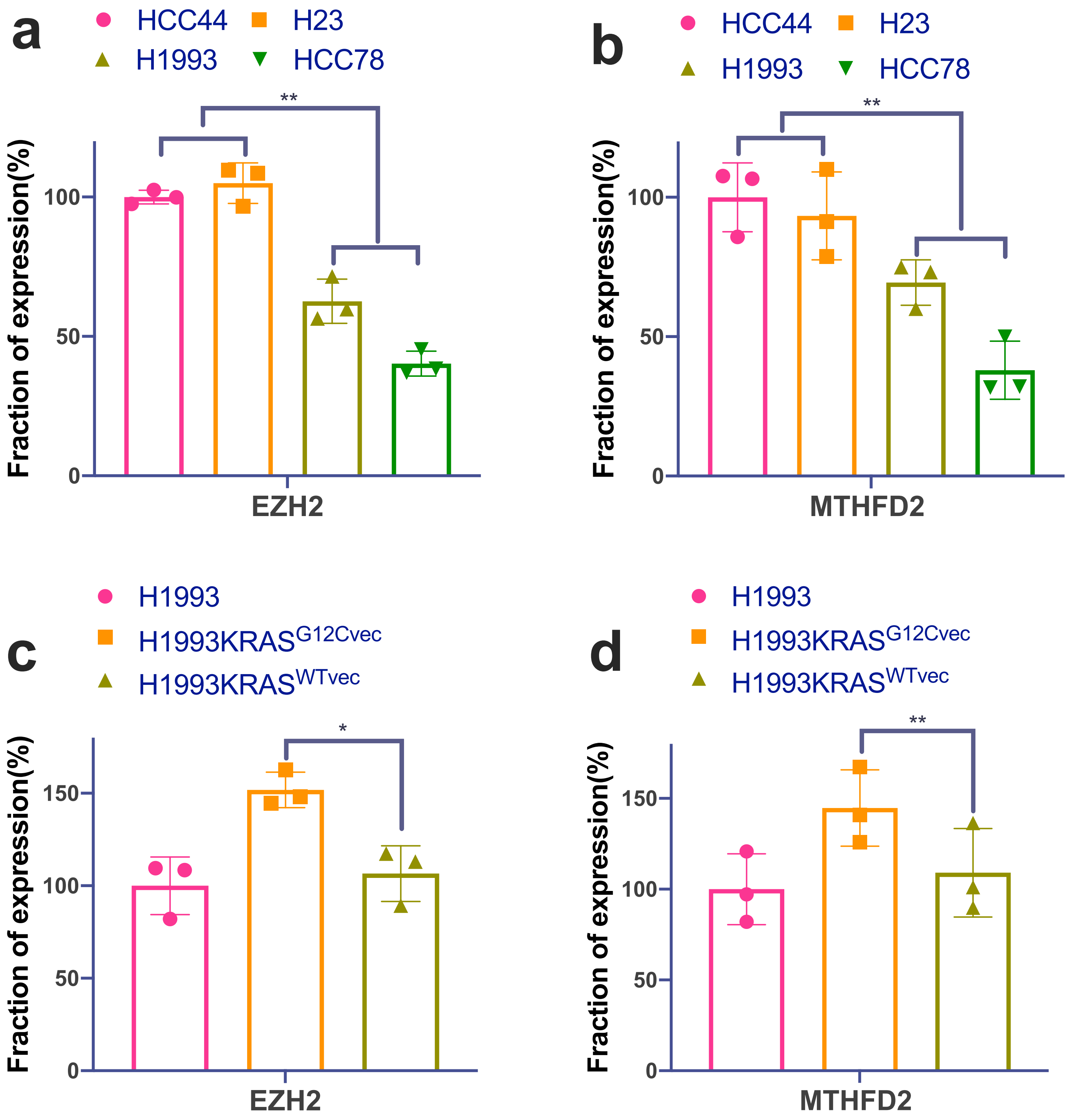

Figure S1 Expression of EZH2 and MTHFD2 in the four AC cell lines HCC44, H23, H1993, and HCC78 and H1993 cells transfected with $Kras^{G12Cvec}$ or $Kras^{WTvec}$ plasmid. Quantification of EZH2 (**a**) and MTHFD2 (**b**) protein expression in the AC cell lines HCC44, H23, H1993,and HCC78. Quantification of EZH2 (**c**) and MTHFD2 (**d**) protein expression in H1993 cells transfected with $Kras^{G12Cvec}$ or $Kras^{WTvec}$ plasmid. GAPDH was used as loading control. Data is depicted as mean ± SEM of at three independent experiments.
