# Supplementary material for: Regulation and Therapeutic Targeting of MTHFD2 and EZH2 in KRAS-Mutated Human Pulmonary Adenocarcinoma
Source: Metabolites. 2022 Jul 15;12(7):652. doi: 10.3390/metabo12070652 (PMC9324032; doi:10.3390/metabo12070652)
Supplement: Supplementary file 1 [file metabolites-12-00652-s001.zip › Li_et_al_Supplementary_Figure S2.pdf]

Figure S2

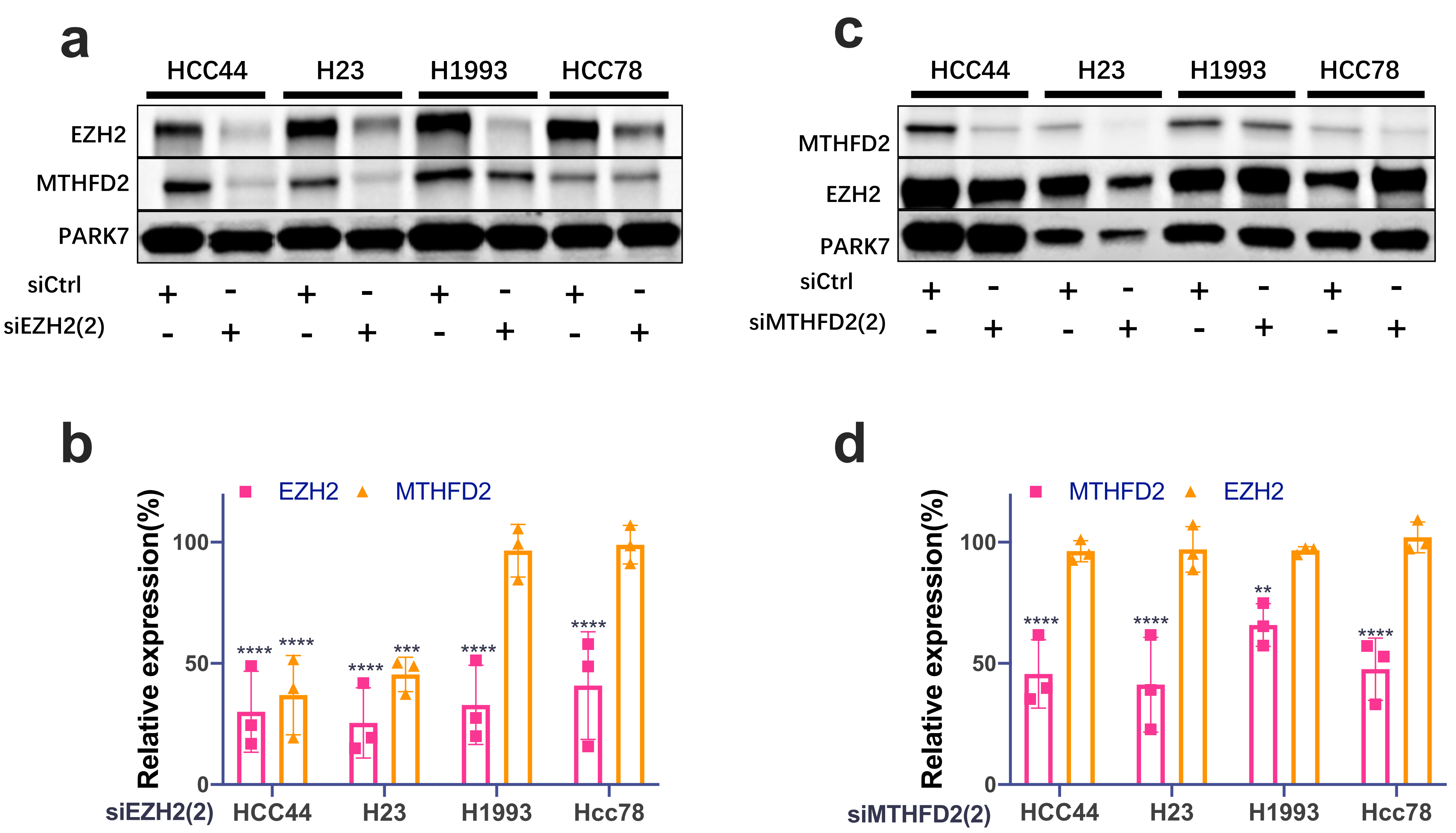

Figure S2 (**a**) Western blot analysis of the four AC cell lines HCC44, H23, H1993, and HCC78 after EZH2 knockdown with siRNA(2). (**b**) Related quantification of EZH2 and MTHFD2 protein expression after EZH2 knockdown. (**c**) Western blot analysis after MTHFD2 knockdown with siRNA(2). (**d**) Related quantification of EZH2 and MTHFD2 protein expression after MTHFD2 knockdown. Data is depicted as mean ± SEM of at three independent experiments.
